# Supplementary material for: A Humanized Yeast Phenomic Model of Deoxycytidine Kinase to Predict Genetic Buffering of Nucleoside Analog Cytotoxicity
Source: Genes (Basel). 2019 Sep 30;10(10):770. doi: 10.3390/genes10100770 (PMC6826991; doi:10.3390/genes10100770)
Supplement: Supplementary file 1 [file genes-10-00770-s001.zip › Additional_Files/Additional_File7_GOTermHeatmaps.zip!/Additional_File7_GOTermHeatmaps/purine_nucleobase_biosynthetic_process.pdf]

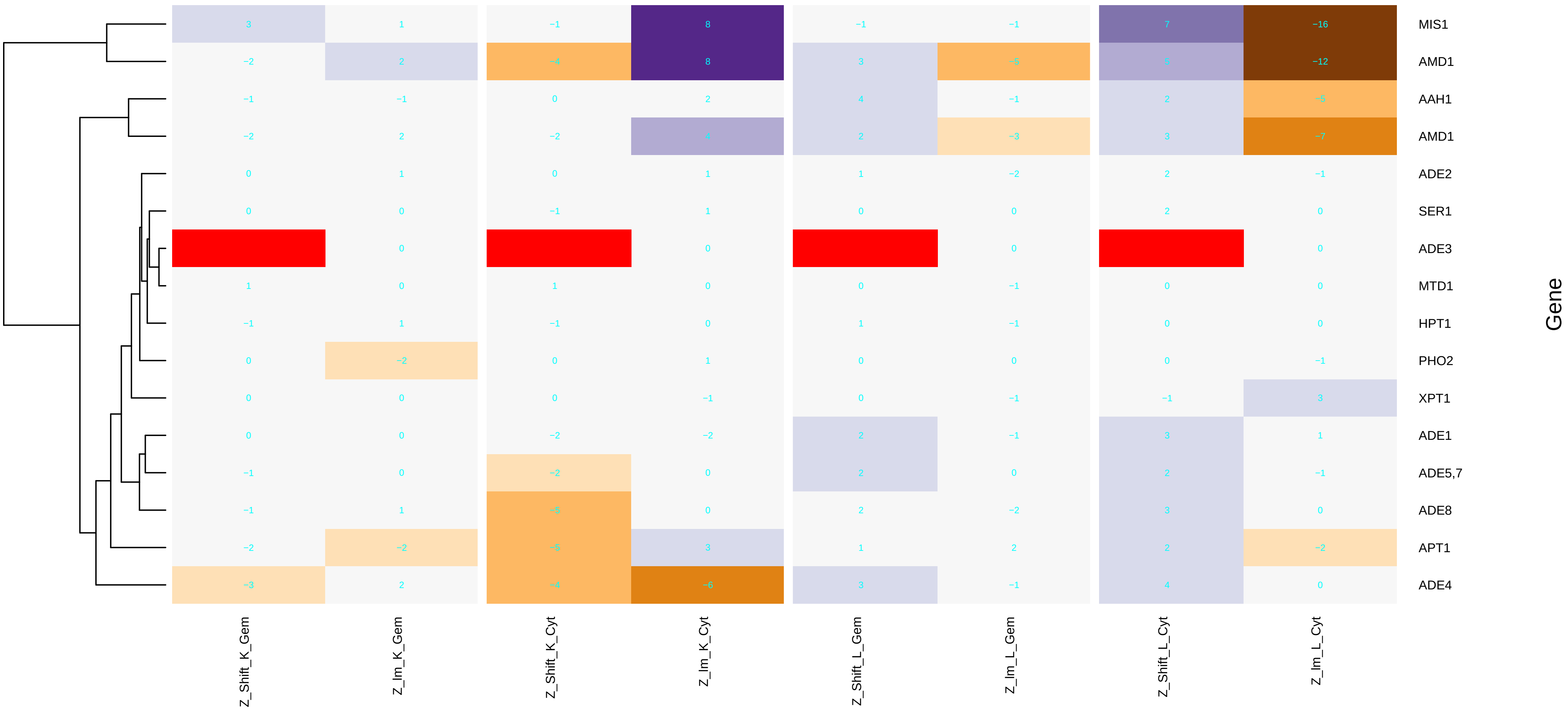
MIS1 3 1 −1 8 −1 −1 7 −16
AMD1 −2 2 −4 8 3 −5 5 −12
AAH1 −1 −1 0 2 4 −1 2 −5
AMD1 −2 2 −2 4 2 −3 3 −7
ADE2 0 1 0 1 1 −2 2 −1
SER1 0 0 −1 1 0 0 2 0
ADE3 0 0 0 0
MTD1 1 0 1 0 0 −1 0 0
HPT1 −1 1 −1 0 1 −1 0 0
PHO2 0 −2 0 1 0 0 0 −1
XPT1 0 0 0 −1 0 −1 −1 3
ADE1 0 0 −2 −2 2 −1 3 1
ADE5,7 −1 0 −2 0 2 0 2 −1
ADE8 −1 1 −5 0 2 −2 3 0
APT1 −2 −2 −5 3 1 2 2 −2
ADE4 −3 2 −4 −6 3 −1 4 0
Z_Shift_K_Gem
Z_Im_K_Gem
Z_Shift_K_Cyt
Z_Im_K_Cyt
Z_Shift_L_Gem
Z_Im_L_Gem
Z_Shift_L_Cyt
Z_Im_L_Cyt
Gene
